# Supplementary material for: A polychromatic ‘greenbeard' locus determines patterns of cooperation in a social amoeba
Source: Nat Commun. 2017 Jan 25;8:14171. doi: 10.1038/ncomms14171 (PMC5288501; doi:10.1038/ncomms14171)
Supplement: Supplementary Information — Supplementary Figures and Supplementary References [file ncomms14171-s1.pdf]

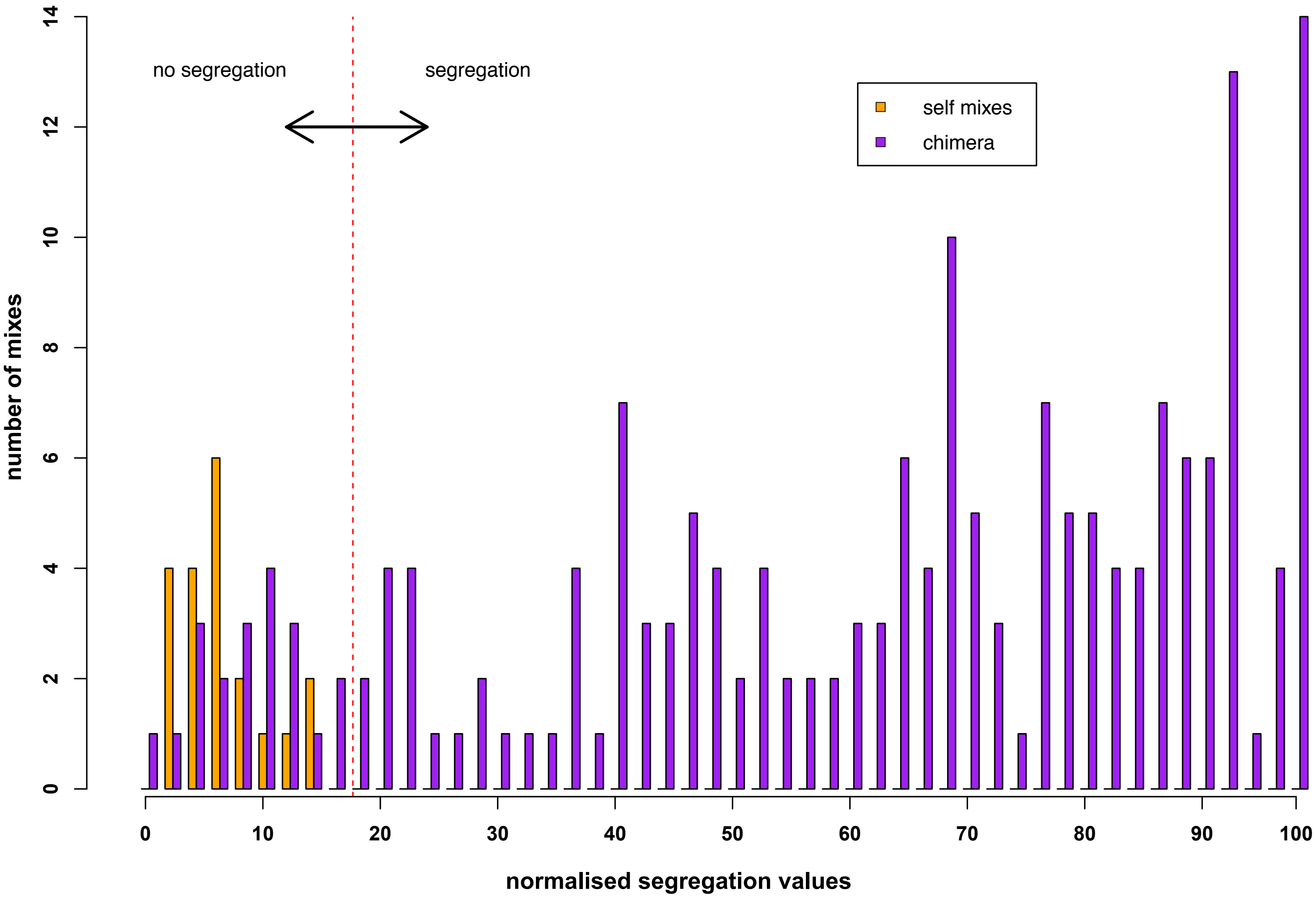

Supplementary Figure 1. Normalised segregation values of self-mixes and chimeras.
A gamma distribution of non-segregation was derived from the self-mixes (orange) and the Šidák [1] corrected cumulative density of 99% (18; red line) used as a cutoff to discriminate between segregation and non-segregation.

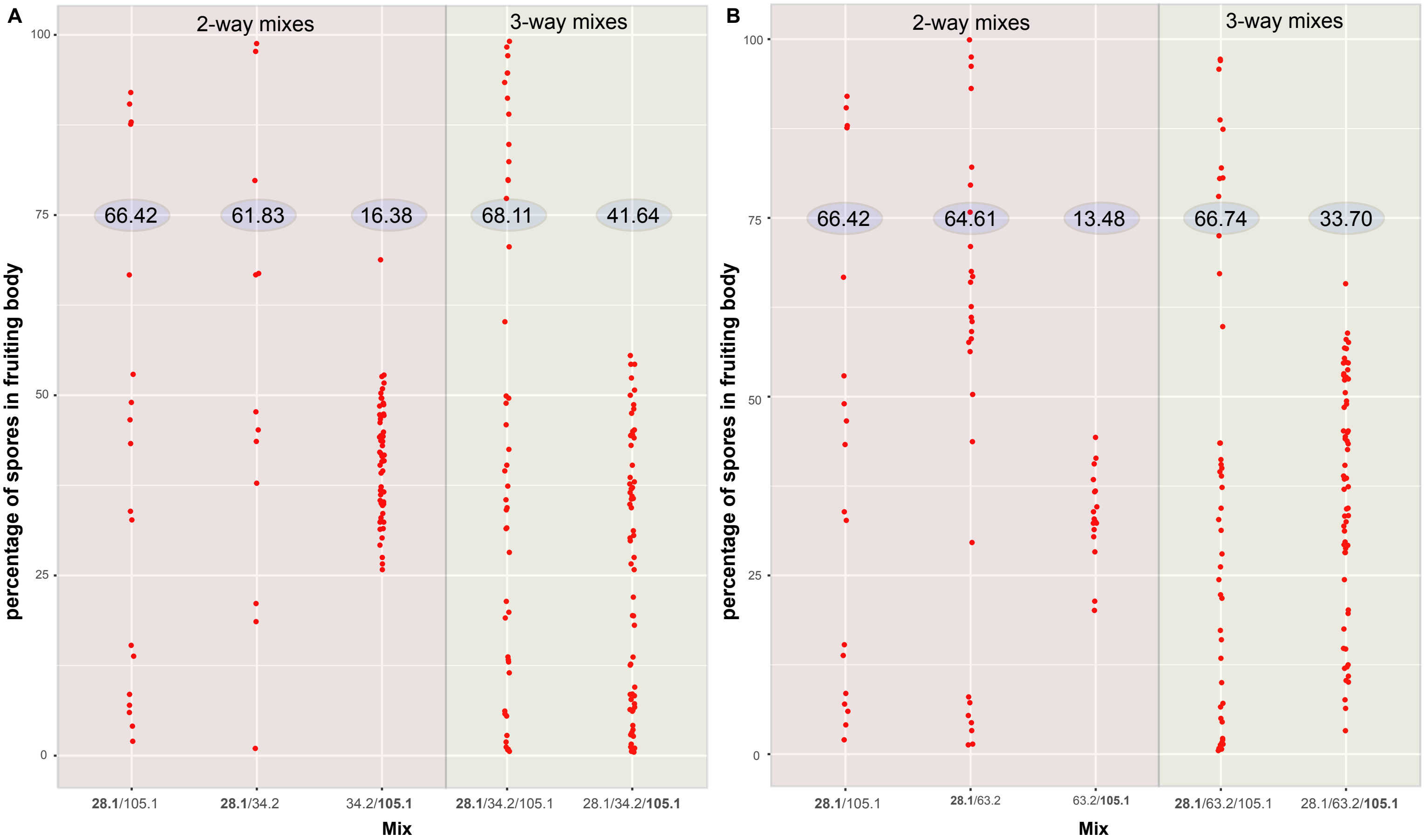

Supplementary Figure 2. Comparison of 2-way and 3-way mixes.

2-way and 3-way mixes of strains are shown. In each case, the labelled strain is highlighted bold.

A. In pairwise mixes, strain NC28.1 segregates strongly from strains NC105.1 (segregation value = 66.42, see 'measuring segregation' in the methods section for a description of how these were calculated) and NC34.2 (seg = 61.83), while strains NC 34.2 and NC105.1 do not segregate (seg = 16.38). When all three strains are mixed and NC28.1 is labelled, the segregation value is the same as pairwise mixes (seg = 68.11) as it segregates from both other strains. If NC105.1 is labelled, an intermediate segregation value compared to pairwise mixes is observed (seg = 41.64) because it segregates from NC28.1 but not from NC34.2.

B. In pairwise mixes, strain NC28.1 segregates strongly from strains NC105.1 (seg = 66.42) and NC63.2 (seg = 64.61), while strains NC 63.2 and NC105.1 do not segregate (13.48). When all three strains are mixed and NC28.1 is labelled, the segregation value is the same as pairwise mixes (seg = 66.74) as it segregates from both other strains. If NC105.1 is labelled, an intermediate segregation value is observed (seg = 33.70) because it segregates from NC28.1 but not from NC63.2.

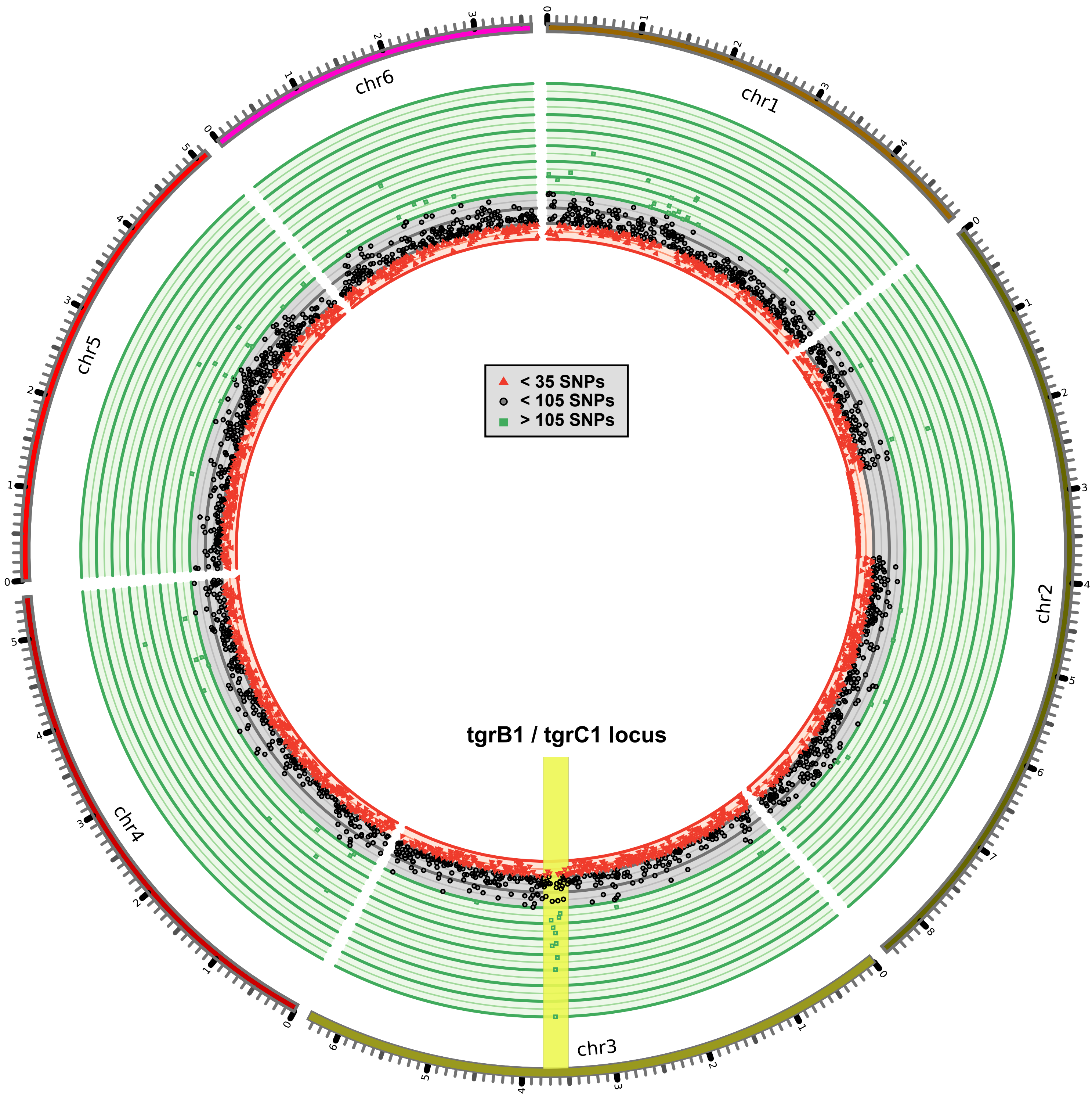

Supplementary Figure 3. Visualization of genome-wide polymorphisms across 20 different strains. SNPs between the 20 wild strains were counted within all 10,000 bp windows and plotted with Circos [2]. Win-dows with less than 35 SNPs are marked with red triangles, windows with 35 – 105 SNPs are marked with grey circles, and windows with more than 105 SNPs are marked with green squares. The maximum number of SNPs per window is 353. This window contains the tgrB1 / tgrC1 locus.

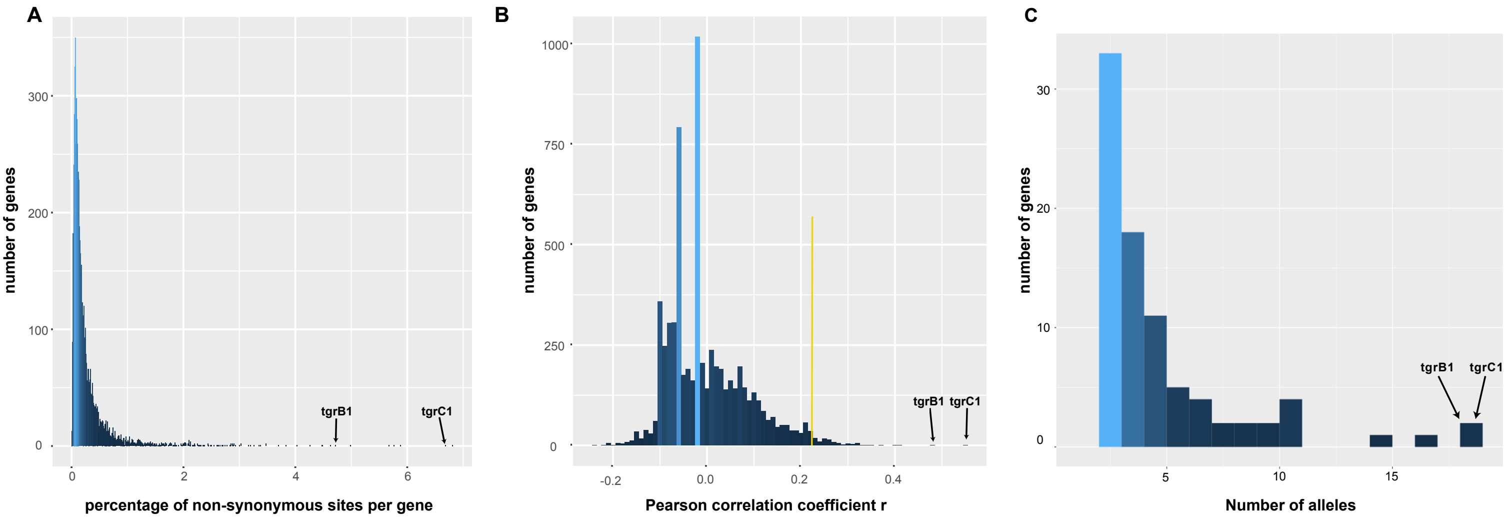

Supplementary Figure 4. Identification of tgrB1 and tgrC1 locus as potential greenbeard locus.
A. Histogram of percentages of non-synonymous sites. 6,532 genes showed at least one non-synonymous site (NS) between the 20 strains. The number of NS was divided by the length of the gene and plotted as a histogram. Values of tgrB1 (4.72 %) and tgrC1 (6.68 %) are marked with arrows.
B. Histogram of correlation coefficients r between pairwise protein distances and segregation values of 6,532 variable genes. Pairwise protein distances were computed using the R package SeqinR [3]. Pearson correlation coefficients were then computed between the pairwise distances and the segregation values for each gene and plotted as a histo-gram. Values of tgrB1 (0.48) and tgrC1 (0.55) are marked with arrows. The majority of the genes show weak correlation between pairwise distances and segregation values but for 85 genes correlation is significant (Pearson's product moment correlation: p-value < 7.85 x 10-6; indicated by yellow line).
C. Histogram of the number of different alleles in 85 greenbeard candidate loci. The number of different alleles was computed for each of the 85 candidate genes. Most of the genes only show two alleles, which makes it impossible for them to create the complex partner specific segregation behaviour. TgrB1 and TgrC1 show the highest number of alleles (18 each).

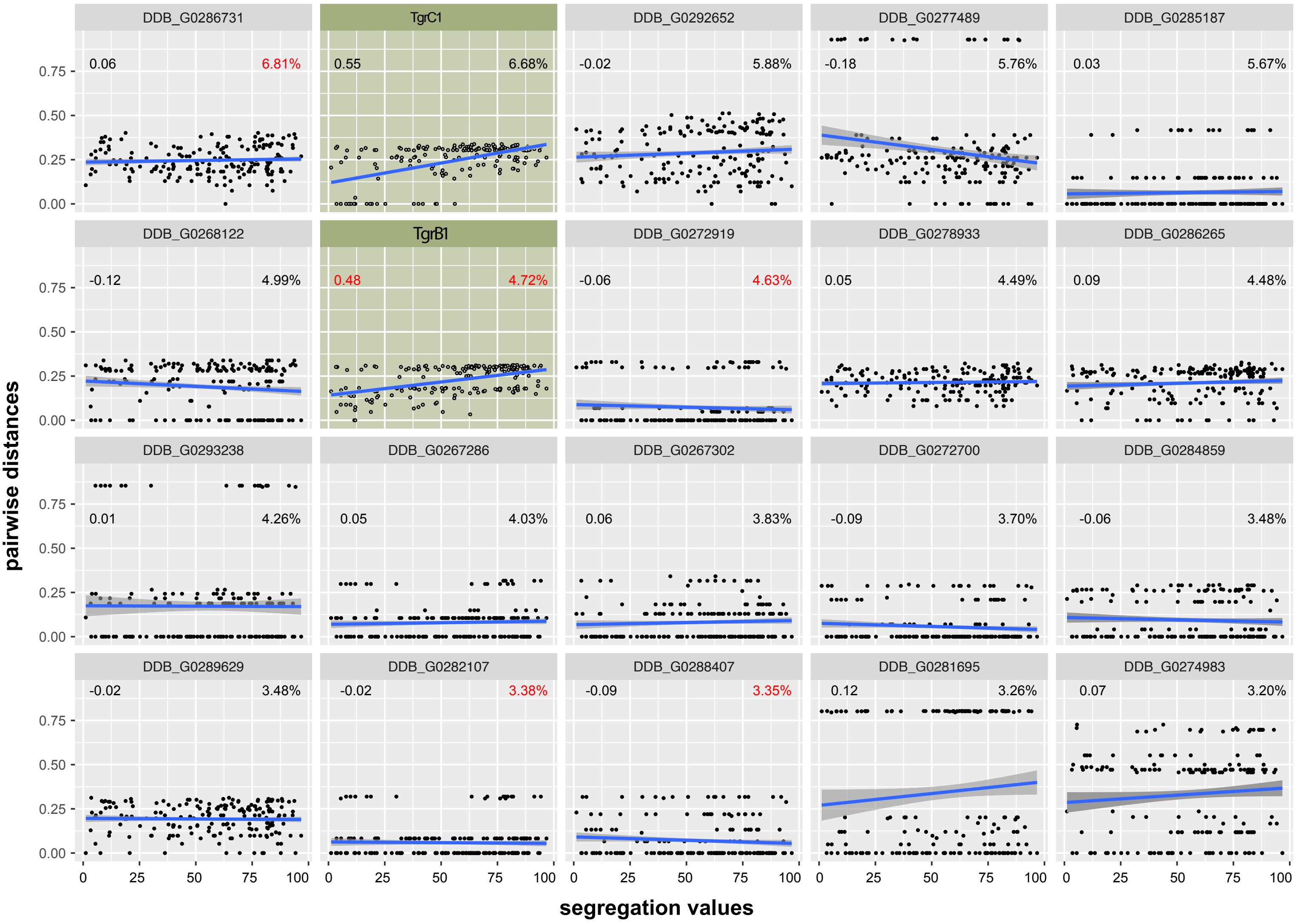

Supplementary Figure 5. Correlation between pairwise sequence distance and segregation values.
For all genes the number of non-synonymous sites was determined using the R package SeqinR [3] and normalized to the length of each gene to get the percentage of non-synonymous sites. Pairwise distances of the 20 genes with the highest percentages (red numbers in each subplot) were plotted against pairwise segregation values. Black numbers in each subplot represent Pearson correlation coefficients, which were also visualized by the blue line with gray shading indicating the 95% confidence envelope. Two (tgrB1, tgrC1; highlighted in green) out of these 20 genes show significant correlation with segregation behaviour.

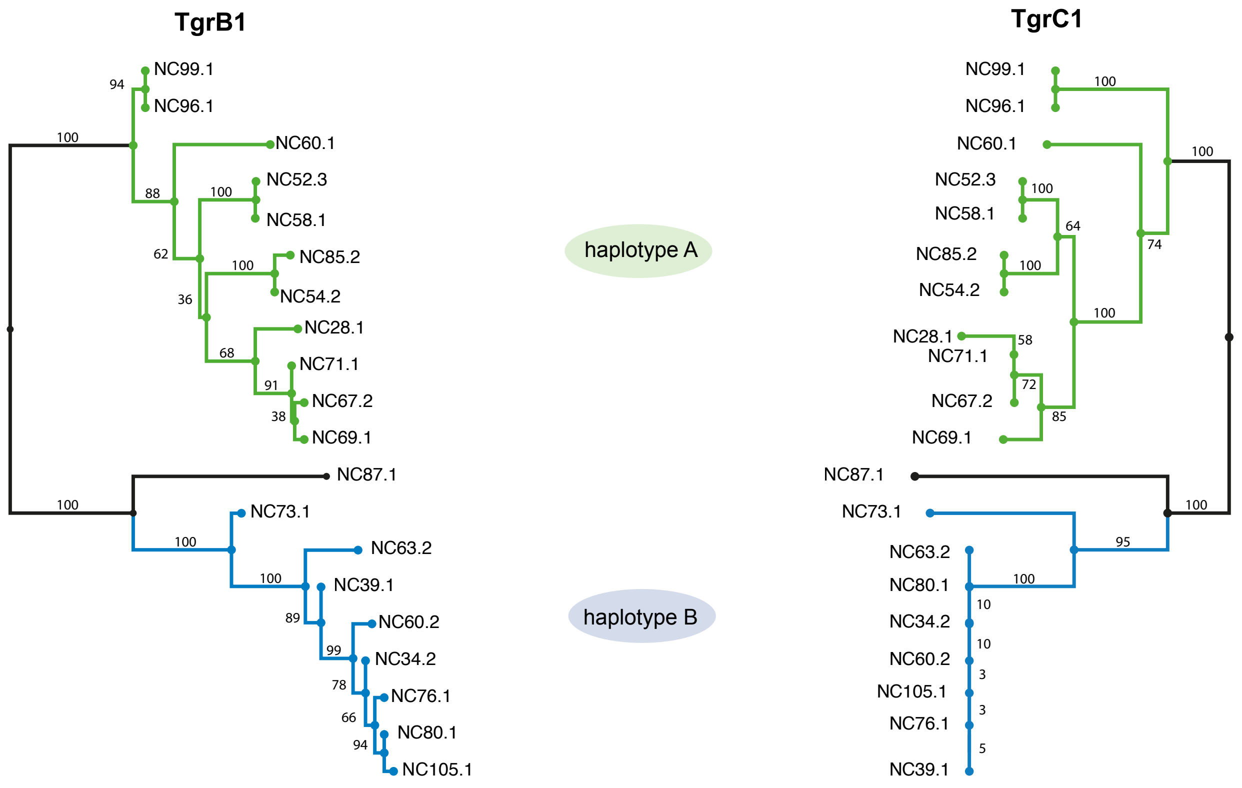

Supplementary Figure 6: Phylogenetic trees of *tgrB1* (left) and *tgrC1* (right).
Nucleotide sequences of *tgrB1* and *tgrC1* from all 20 strains were aligned using ClustalW. The alignments were then converted to phylip format and phylogenetic trees computed using PhyML using the HKY85 model, estimated number of invariant sites and gamma shape parameter, best of NNI and SPR moves, and bootstrapping (100 repeats). Numbers on the branches correspond to bootstrap values. Haplotype groups are highlighted in green (haplotype A) and blue (haplotype B).

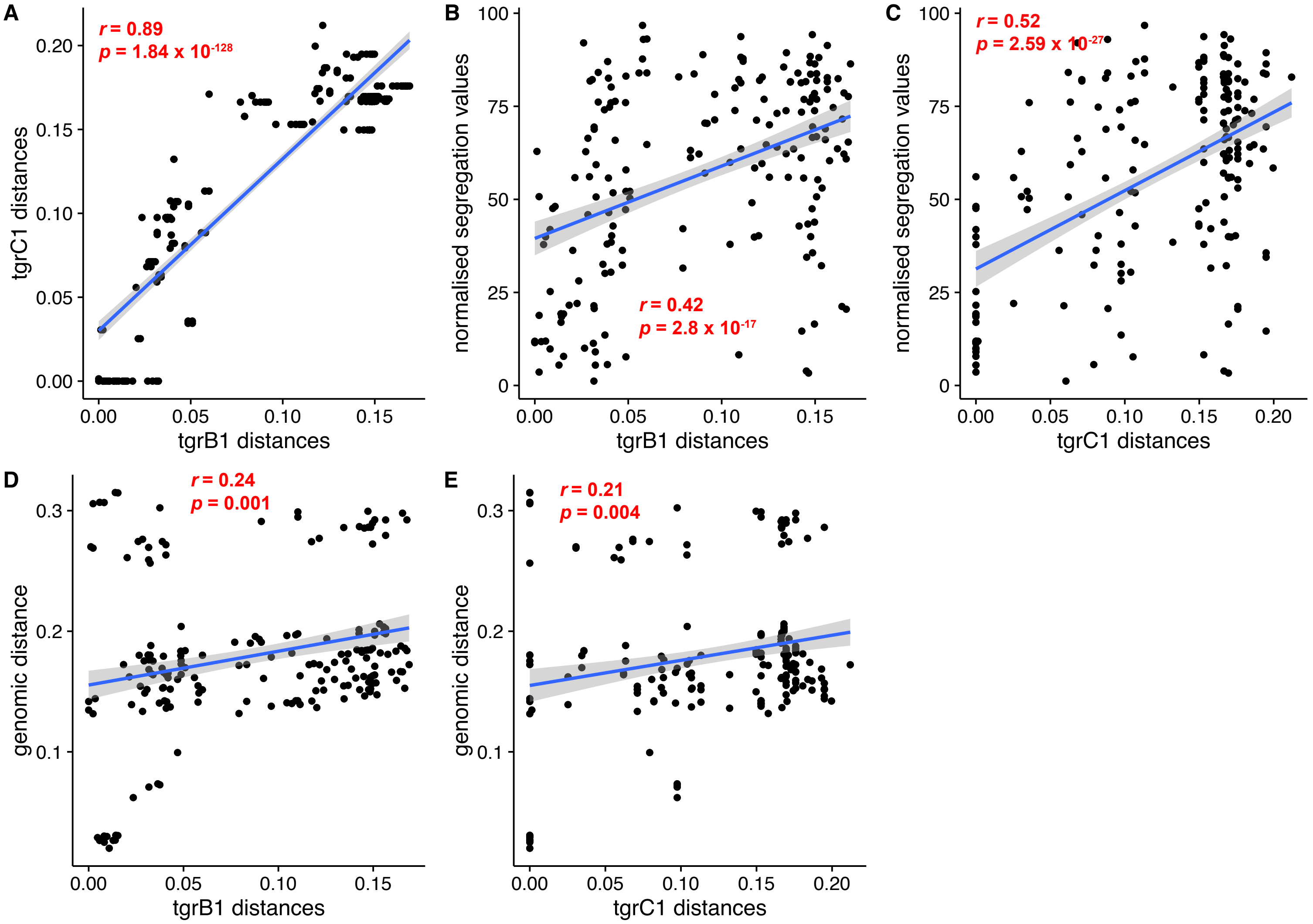

Supplementary Figure 7. Relationships between segregation values, genomic, and tree distances.
TgrB1 distances plotted against TgrC1 distances (A), segregation values plotted against TgrB1 (B) and TgrC1 (C) distances, genomic distances plotted against TgrB1 (D) and TgrC1 (E) distances. Correlations between all pairings (visualized by the blue line, with gray shading indicating the 95% confidence envelope) are positive and highly significant, depicted by the red numbers.

A

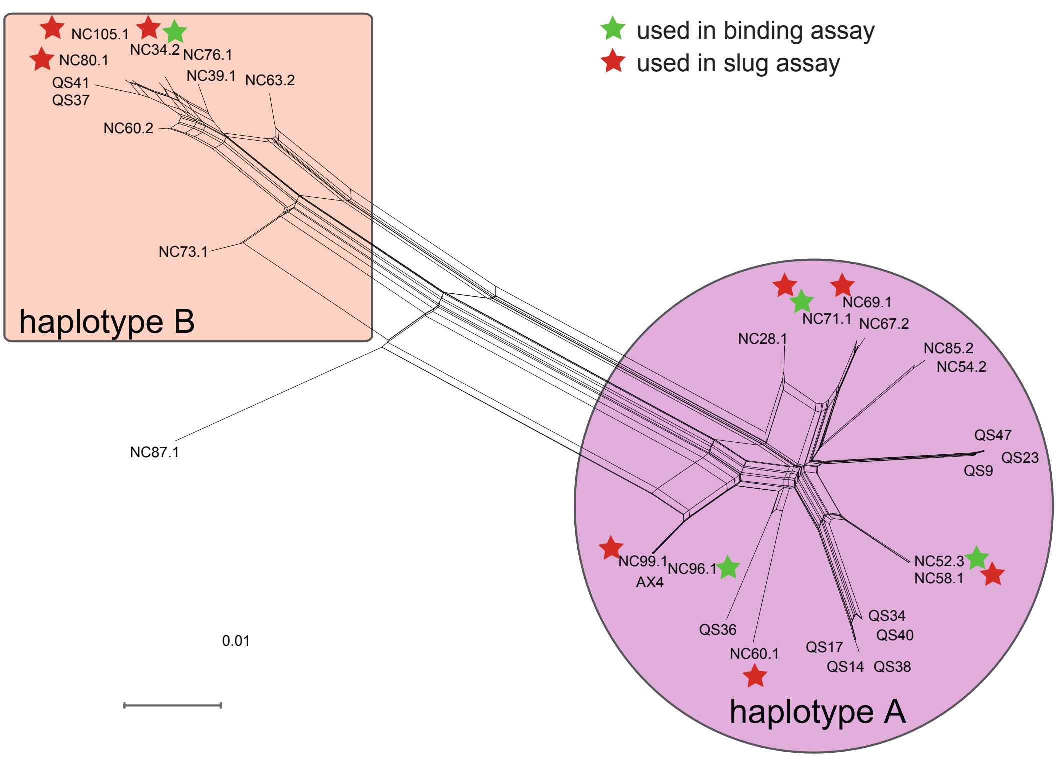

B

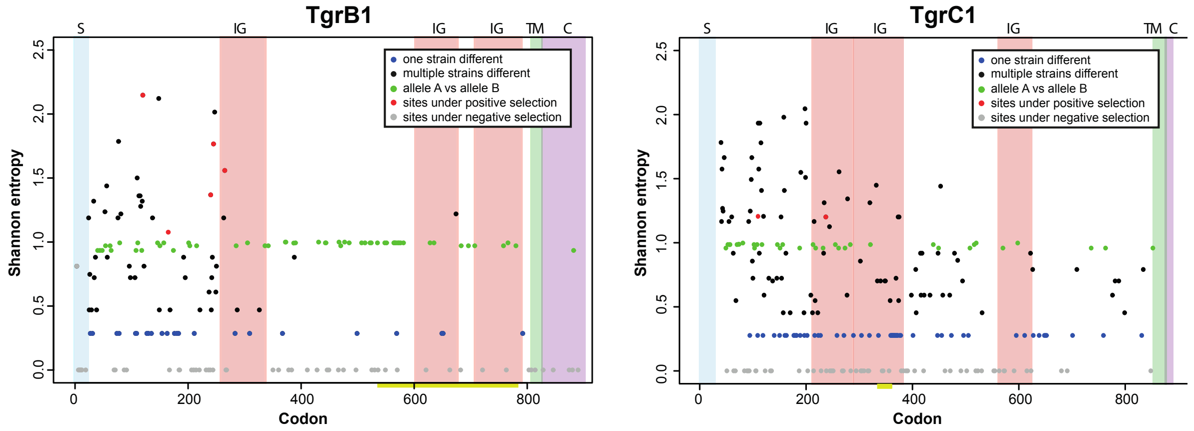

Supplementary Figure 8. Variability of tgr genes.
A. Phylogenetic network of TgrB1 protein alignment containing 20 NC, eleven QS strains and AX4. TgrB1 protein sequences of eleven previously analysed wild strains as well as AX4 from NCBI were aligned together with the 20 sequences of the NC strains. A phylogenetic network was computed using SplitsTree4 [4]. Green stars depict strains chosen for the binding assay while red stars indicate strains used in slug migration assays. Strains with haplotypes A and B are highlighted in red and purple respectively.
B. Normalised Shannon entropy across all amino acids in TgrB1 (left) and TgrC1 (right) protein alignments. Assembled tgrB1 and tgrC1 coding sequences for all 20 strains were translated and aligned using ClustalW [5,6]. The Shannon entropy score was computed for each position in the alignments using the bio3d R package [7-9]. Invariable positions have an entropy score of zero and were excluded from the plot. Blue dots depict all positions where one strain has a different amino acid than all other strains, black dots depict positions with multiple differences, green dots depict positions with eight strains showing one amino acid (haplotype A), while eleven other strains show a different amino acid (haplotype B). Red dots illustrate positions found to be under significant diversifying selection using the HyPhy package [10,11] and the MEME algorithm [12]. For both proteins six domains were predicted using Interpro [13]: S (TgrB1: M1 - S24; TgrC1: M1 – A28) depicts signal peptides (blue), three Ig-like (IPR013783; TgrB1: G258 – V337, K602 – S678, S708 – E791; TgrC1: I212 - I288, C290 – I382, T561 – G623; red) and one transmembrane domain (TM; M808 – A826; L853 – F875; green). The C-terminal part (C; K827-D902; K876– F889) is predicted to be cytoplasmic (purple). The regions marked by yellow bars (K537 - A783; I336 – V360) show regions found to be sufficient to mediate TgrB-TgrC protein interactions [14]. Within haplotype variation is clustered at the N-terminus of both proteins, while the predicted binding domains only show between haplotype polymorphisms.

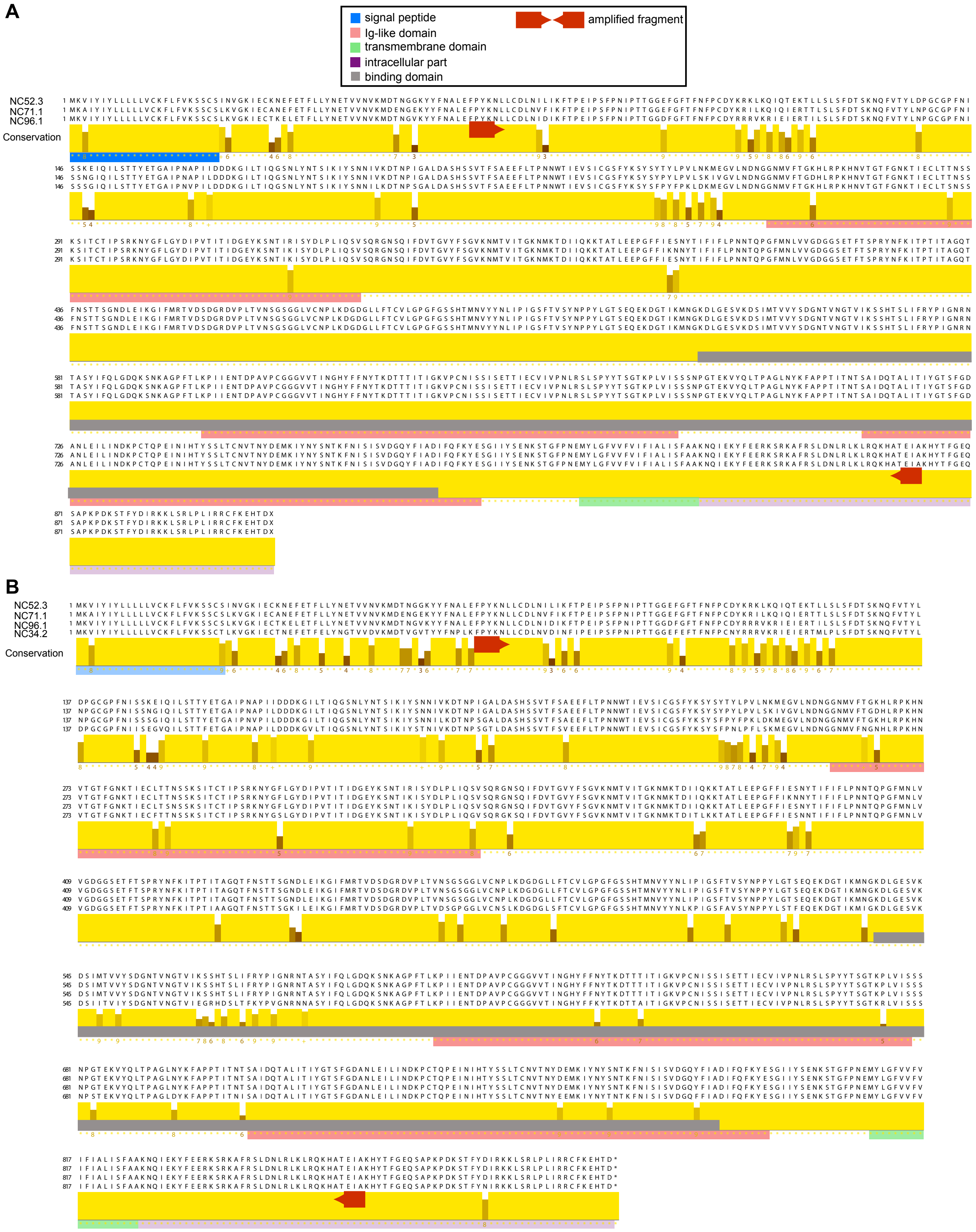

Supplementary Figure 9. Full length alignments of TgrB1.

A. Graphical representation of TgrB1 using strains NC 52.3, NC71.1, and NC96.1. TgrB1 sequences of three strains from haplotype group A were aligned using ClustalW. The yellow bar graph underneath the sequences represents the conservation at each site. Variable sites containing amino acids with very different properties have low and dark bars, while conserved sites have a high and yellow bar. Domains are annotated as described in Supplementary Fig. 8B. Red arrows depict the primers used for the amplification of a nearly full length (88.79 %) protein. Sequences are identical after N389.

B. Graphical representation of TgrB1 using strains NC 52.3, NC71.1, NC96.1, and NC34.2. TgrB1 sequences of three strains from haplotype group A and one strain from haplotype group B were aligned using ClustalW. The yellow bar graph underneath the sequences represents the conservation at each site. Variable sites containing amino acids with very different properties have low and dark bars, while conserved sites have a high and yellow bar. Domains are annotated as described in Supplementary Figure 8B. Red arrows depict the primers used for the amplification of a nearly full length (88.79 %) protein. Sequences show polymorphisms throughout the protein.

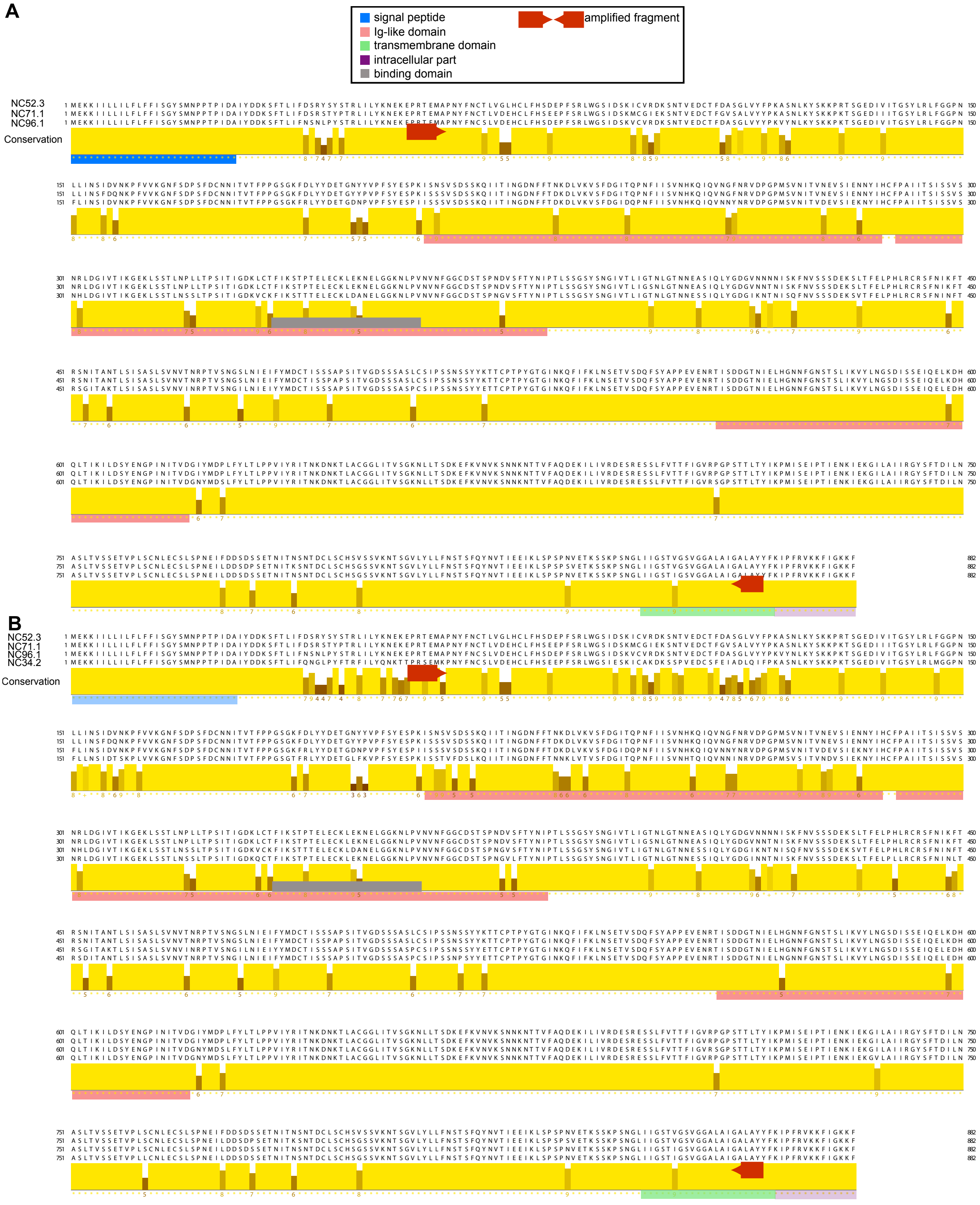

Supplementary Figure 10. Full length alignments of TgrC1.

A. Graphical representation of TgrC1 using strains NC 52.3, NC71.1, and NC96.1. TgrC1 sequences of three strains from haplotype group A were aligned using ClustalW. The yellow bar graph underneath the sequences represents the conservation at each site. Variable sites containing amino acids with very different properties have low and dark bars, while conserved sites have a high and yellow bar. Domains are annotated as described in Supplementary Fig. 8B. Red arrows depict the primers used for the amplification of a nearly full length (98.5 %) protein.

B. Graphical representation of TgrC1 using strains NC 52.3, NC71.1, NC96.1, and NC34.2. TgrC1 sequences of three strains from haplotype group A and one strain from haplotype group B were aligned using ClustalW. The yellow bar graph underneath the sequences represents the conservation at each site. Variable sites containing amino acids with very different properties have low and dark bars, while conserved sites have a high and yellow bar. Domains are annotated as described in Supplementary Figure 8B. Red arrows depict the primers used for the amplification of a nearly full length (98.5 %) protein. Sequences show polymorphisms throughout the protein.

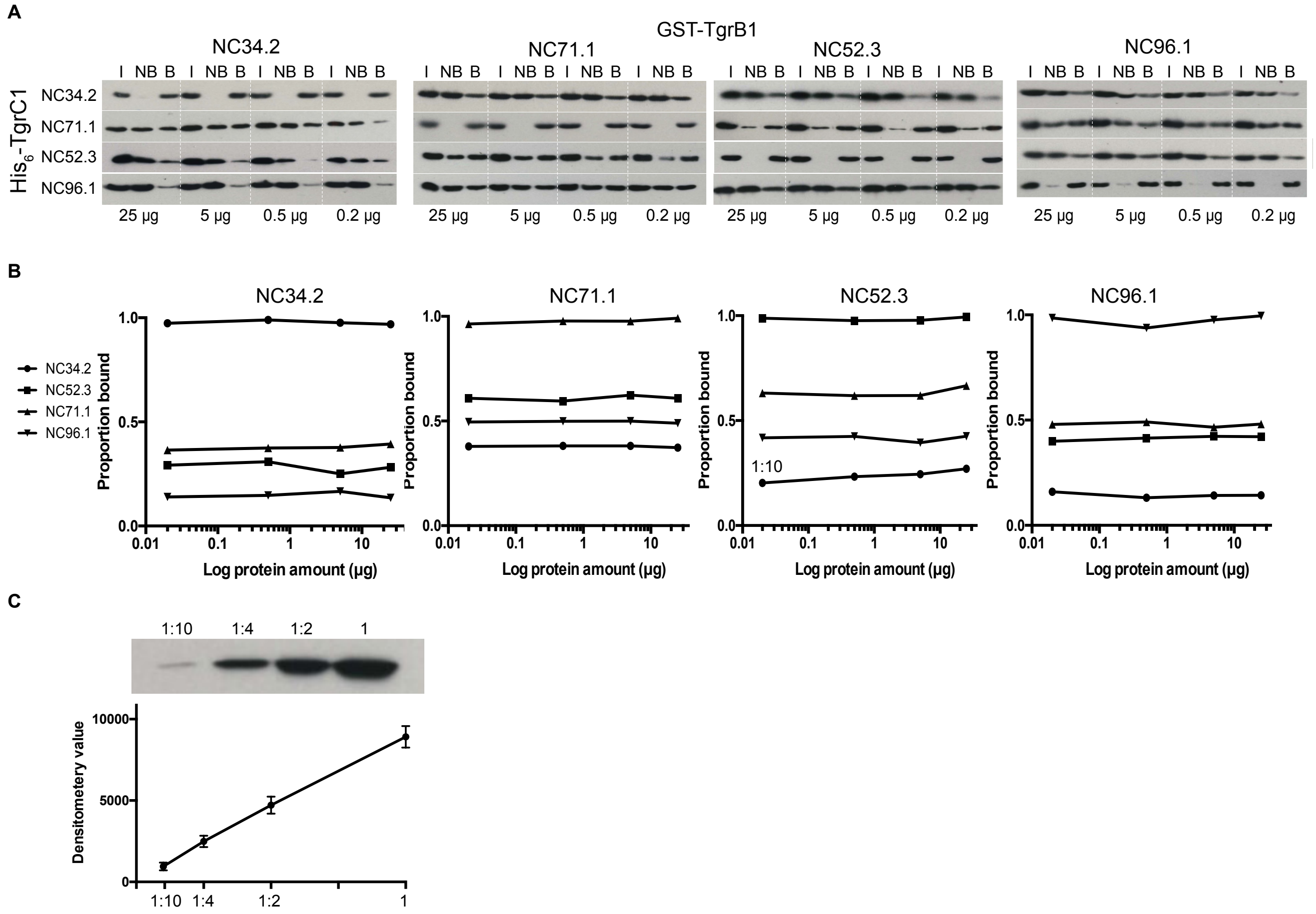

Supplementary Figure 11. Protein interactions are robust to changes in protein concentration.

A. Immunoprecipitation of bacterially expressed His6 – TgrC1 and GST - TgrB1 at different protein concentrations. His6 – TgrC1 was incubated with GST - TgrB1 from each strain. His6 – TgrC1 complexes were isolated and any bound GST - TgrB1 was detected with an anti GST antibody (I = input protein, NB = not bound protein and B = bound protein). Four different protein amounts were tested (25 µg, 5 µg, 0.5 µg, or 0.2 µg). After immunoprecipitation, samples were normalised by dilution to ensure that equal amounts were loaded for quantification. The strength of binding was quantified by comparing the relative amount of protein (band intensity) in the bound and not bound fractions.

B. Quantification of protein binding. The strength of protein binding between Tgr proteins from the different strains is unaffected by protein concentration.

C. All densitometry measurements are in the linear range in all protocols used. Top panel: 25µg of GST-TgrB1 protein was loaded neat, at a 1:2 dilution or a 1:4 dilution or 1:10 dilution and detected by Western blotting using anti-GST antibody. Bottom panel: Densitometry analyses of GST-TgrB1 compared to protein concentration. Error bars represent s.e.m. from three independent GST-TgrB1 protein preparations.

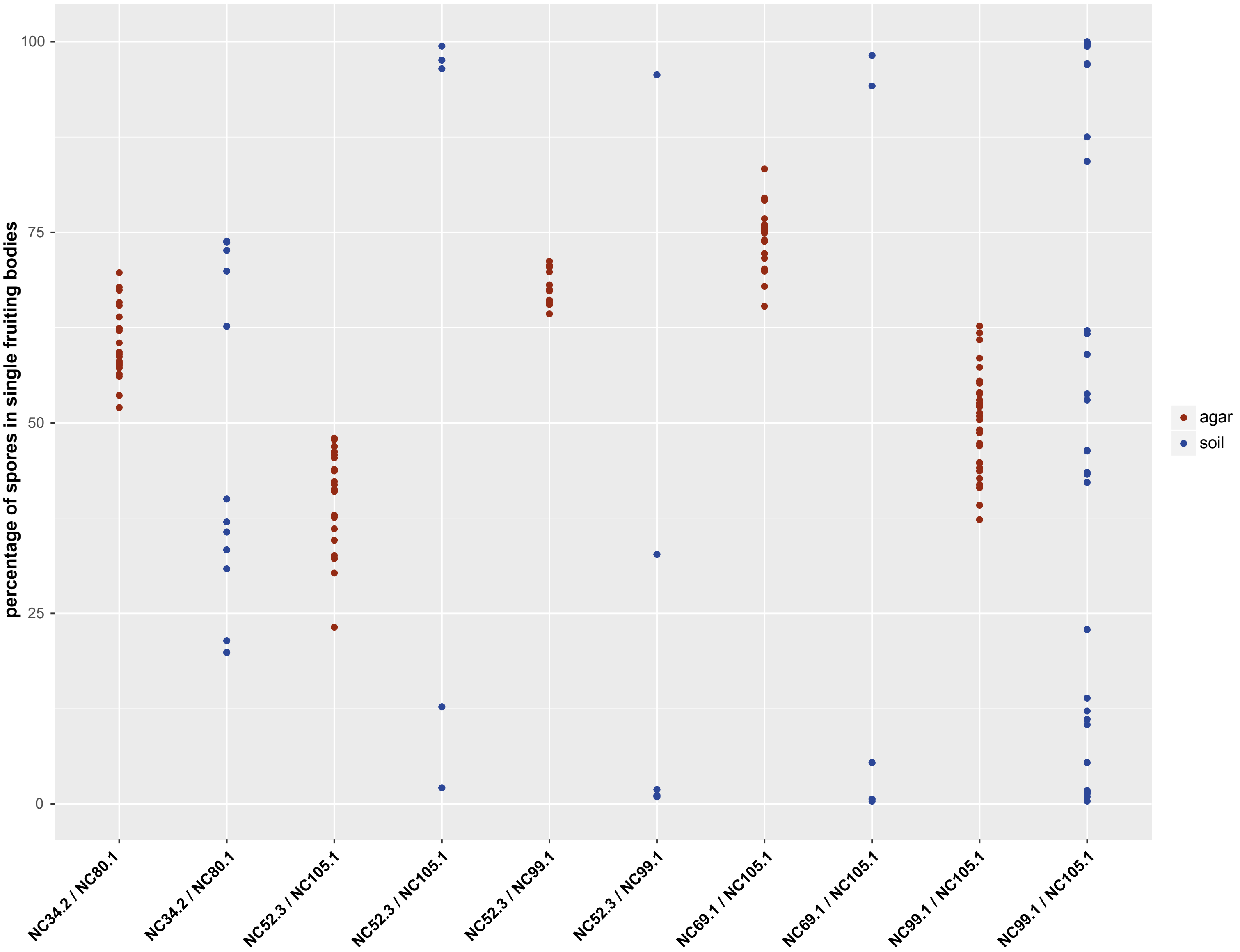

Supplementary Figure 12. Segregation is mostly eliminated on an agar substrate.
Mixes of strains NC34.2, NC80.1, NC52.3, NC105.1, NC99.1, and NC69.1 were allowed to develop on soil and agar, where one strain of each mix was labelled. Points show percentage of spores of the labelled strain on agar (red) and soil (blue). Variation across fruiting bodies that developed on agar is significantly lower than those that developed on soil (F – test, p < 2.2 x 10-16).

Figure 2B

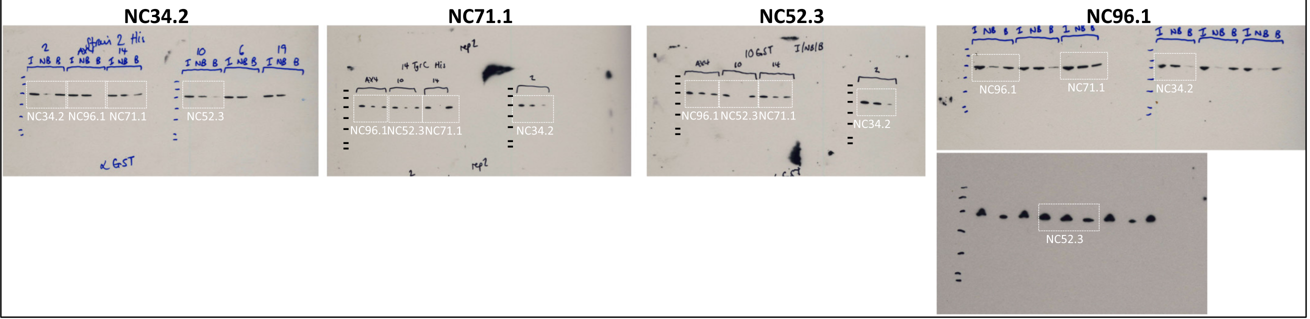

Supplementary Figure 11A

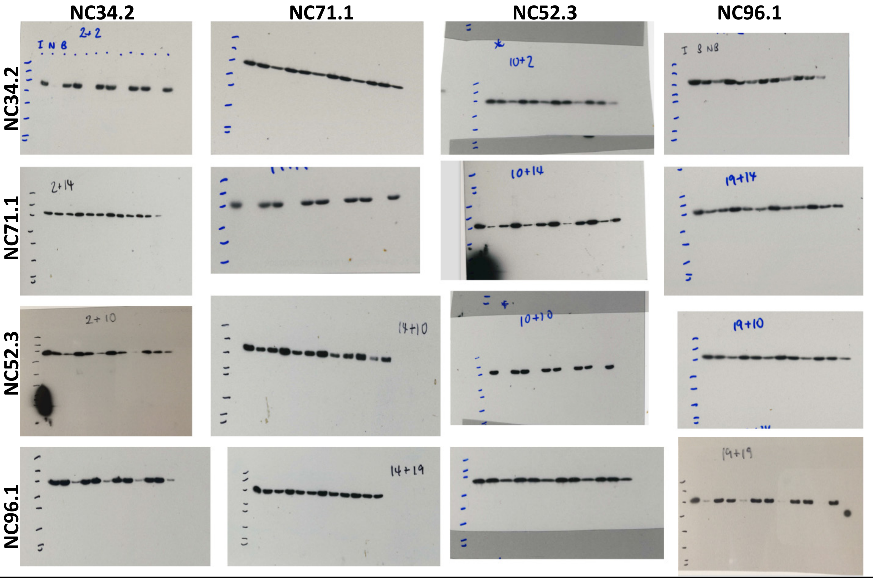

Supplementary Figure 11C

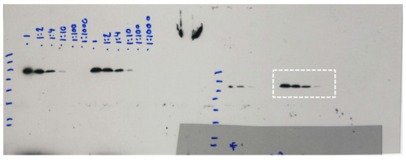

Supplementary Figure 13. Uncropped westerns of Figure 2B, Supplementary Figure 11A, and Suppementary Figure 11C.

Supplementary References

1 Šidák, Z. Rectangular confidence regions for the means of multivariate normal distributions. Journal of the American Statistical Association 62, 626-633, (1967).

2 Krzywinski, M. *et al*. Circos: an information aesthetic for comparative genomics. Genome research 19, 1639-1645, (2009).

3 Charif, D. & Lobry, J. R. in *Structural approaches to sequence evolution* 207-232 (Springer, 2007).

4 Huson, D. H. & Bryant, D. Application of phylogenetic networks in evolutionary studies. Molecular Biology and Evolution 23, 254-267, (2006).

5 Thompson, J. D., Higgins, D. G. & Gibson, T. J. CLUSTAL W: improving the sensitivity of progressive multiple sequence alignment through sequence weighting, position-specific gap penalties and weight matrix choice. Nucleic Acids Research 22, 4673-4680, (1994).

6 Larkin, M. a. *et al*. Clustal W and Clustal X version 2.0. Bioinformatics 23, 2947-2948, (2007).

7 Mirny, L. a. & Shakhnovich, E. I. Universally conserved positions in protein folds: reading evolutionary signals about stability, folding kinetics and function. Journal of Molecular Biology 291, 177-196, (1999).

8 Grant, B. J., Rodrigues, A. P. C., ElSawy, K. M., McCammon, J. A. & Caves, L. S. D. Bio3d: An R package for the comparative analysis of protein structures. Bioinformatics 22, 2695-2696, (2006).

9 Shannon, C. E. A mathematical theory of communication. The Bell System Technical Journal 27, 379-423, (1948).

10 Delport, W., Poon, A. F. Y., Frost, S. D. W. & Kosakovsky Pond, S. L. Datamonkey 2010: A suite of phylogenetic analysis tools for evolutionary biology. Bioinformatics 26, 2455-2457, (2010).

11 Kosakovsky Pond, S. L. & Frost, S. D. W. Datamonkey: Rapid detection of selective pressure on individual sites of codon alignments. Bioinformatics 21, 2531-2533, (2005).

12 Murrell, B. *et al*. Detecting individual sites subject to episodic diversifying selection. PLoS Genetics 8, (2012).

13 Mitchell, a. *et al*. The InterPro protein families database: the classification resource after 15 years. Nucleic Acids Research 43, D213-D221, (2014).

14 Chen, G. *et al*. TgrC1 mediates cell-cell adhesion by interacting with TgrB1 via mutual IPT/TIG domains during development of *Dictyostelium discoideum*. Biochemical Journal, (2013).
